# Risk of New-Onset Atrial Fibrillation Post-cavotricuspid Isthmus Ablation in Typical Atrial Flutter Without History of Atrial Fibrillation

**DOI:** 10.3389/fphys.2021.763478

**Published:** 2021-11-30

**Authors:** Jia-hui Li, Hai-yang Xie, Yan-qiao Chen, Zhong-jing Cao, Qing-hui Tang, Xiao-gang Guo, Qi Sun, Jian Ma

**Affiliations:** Arrhythmia Center, State Key Laboratory of Cardiovascular Disease, Fuwai Hospital, National Center for Cardiovascular Diseases, Chinese Academy of Medical Sciences and Peking Union Medical College, Beijing, China

**Keywords:** typical atrial flutter, atrial fibrillation, catheter ablation, advanced interatrial block (aIAB), left atrial diameter (LAD), CHA2DS2-VASc score

## Abstract

**Aims:** The aim was to describe the incidence of atrial fibrillation (AF) after cavotricuspid isthmus (CTI) ablation in patients with typical atrial flutter (AFL) without history of AF and to identify risk factors for new-onset AF after the procedure.

**Methods:** A total of 191 patients with typical AFL undergoing successful CTI ablation were enrolled. Patients who had history of AF, structural heart disease, cardiac surgery, or ablation or who received antiarrhythmic drug after procedure were excluded. Clinical and electrophysiological data were collected.

**Results:** There were 47 patients (24.6%) developing new AF during a follow-up of 3.3 ± 1.9 years after CTI ablation. Receiver operating characteristic (ROC) curves indicated that the cut-off values of left atrial diameter (LAD) and CHA_2_DS_2_-VASc score were 42 mm and 2, with area under the curve of 0.781 and 0.550, respectively. The multivariable Cox regression analysis revealed that obstructive sleep apnea (OSA) [hazard ratio (HR) 3.734, 95% confidence interval (CI) 1.470–9.484, *P* = 0.006], advanced interatrial block (aIAB) (HR 2.034, 95% CI 1.017–4.067, *P* = 0.045), LAD > 42 mm (HR 2.710, 95% CI 1.478–4.969, *P* = 0.001), and CHA_2_DS_2_-VASc score > 2 (HR 2.123, 95% CI 1.118–4.034, *P* = 0.021) were independent risk factors of new-onset AF.

**Conclusion:** A combination of OSA, aIAB, LAD > 42 mm, and CHA_2_DS_2_-VASc > 2 was a strongly high risk for new-onset AF after ablation for typical AFL, and it had significance in postablation management in clinical practice.

## Introduction

Catheter ablation of cavotricuspid isthmus (CTI) is an effective procedure with high success rate for typical atrial flutter (AFL). The development of atrial fibrillation (AF) has always been a big concern after the ablation, and prediction of AF occurrence is important to optimize postablation management of AFL. Previous studies have demonstrated an incidence of AF ranging from 25 to 82% in patients with typical AFL after CTI ablation (Chinitz et al., 2007; Ellis et al., 2007; Voight et al., 2014; Chen et al., 2015; Celikyurt et al., 2017; Fujimoto et al., 2020). However, in most of these studies, patients with and without prior AF were both enrolled, and the history of AF was identified as the greatest risk factor for AF occurrence during the postablation period. Data on incidence and predictors of AF in patients without history of AF after typical AFL ablation are still relatively sparse.

The aim of this study was (a) to describe the incidence of AF after CTI ablation in patients with typical AFL without history of AF and (b) to identify risk factors for the occurrence of AF after CTI ablation.

## Materials and Methods

### Study Population

**FIGURE 1:**
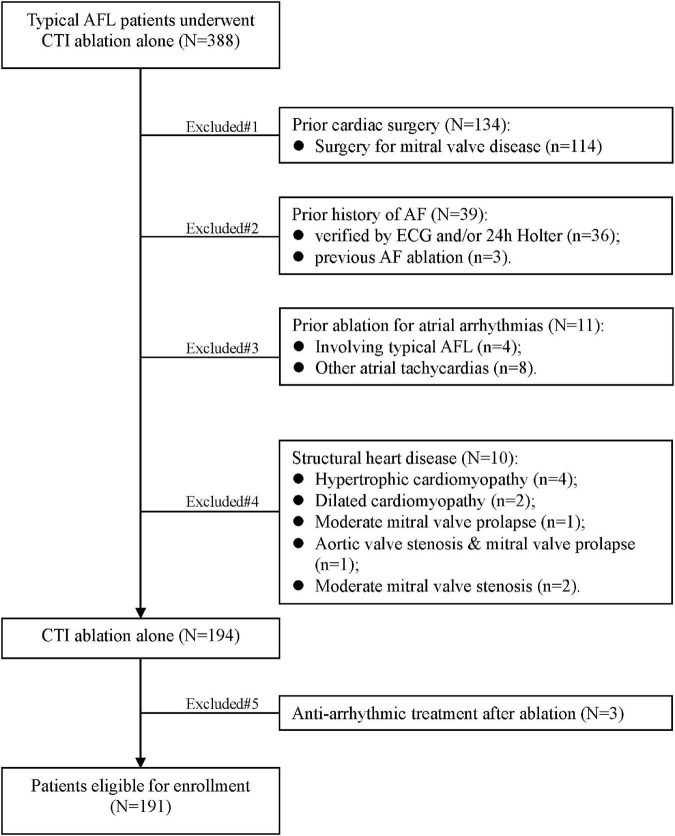
Flowchart of this study. AF, atrial fibrillation; AFL, atrial flutter; CTI, cavotricuspid isthmus.

This study involved 388 patients who were first diagnosed as typical AFL and underwent CTI ablation alone at Ward 2 of Arrhythmia Center between May 2012 and January 2020. All medical records, 12-lead electrocardiogram (ECG), and 24-h Holter obtained from enrolled patients were reviewed to exclude any atrial arrhythmias before the onset of flutter. Of the 39 patients excluded for prior history of AF, 36 had AF verified by ECG and/or 24-h Holter, and the other 3 had previous AF ablation reported by medical records. Other excluded subjects were those with previous history of cardiac surgery (*N* = 134), ablation for atrial arrhythmias (*N* = 11), structural heart disease (*N* = 10), and treatment with antiarrhythmic drugs (AADs) after ablation (*N* = 3). A total of 191 patients with typical AFL confirmed by ECG, Holter, or electrophysiological study were enrolled in final study (Figure 1). The data on baseline characteristics, echocardiographic, and electrocardiographic parameters before ablation were recorded. CHA_2_DS_2_-VASc score was calculated as previously (Liu et al., 2020) introduced. The study was conducted in accordance with the Declaration of Helsinki (as revised in 2013). The study was approved by the ethics committee of Fuwai Hospital, and informed consent was obtained from all the patients.

### Catheter Ablation

Typical AFL was defined as regular negative sawtooth flutter waves in the inferior leads and positive flutter waves in lead V1, with a regular atrial rate between 240 and 350 beats/min. Entrainment techniques were used to determine CTI dependency. Radiofrequency ablation lesions were performed from the tricuspid valve to the inferior vena cava. The successful ablation was defined by the bidirectional CTI block after a surveillance period of at least 10 min. Inducibility of any atrial arrhythmia after CTI ablation was attempted at the discretion of the operator, using atrial burst pacing with infusion of isoproterenol. Induced AF that lasted over 30 s was defined as sustained AF. If AF could not spontaneously convert to sinus rhythm, the electrical cardioversion would be performed.

### Electrophysiology Study

**FIGURE 2:**
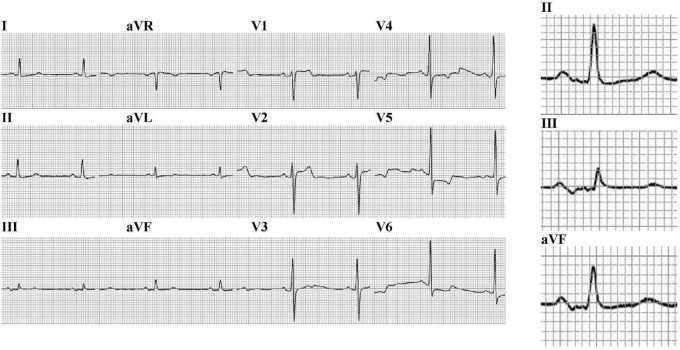
The 12-lead electrocardiogram of a patient with advanced interatrial block (P-wave duration > 120 ms with a biphasic morphology in inferior leads) (25 mm/s, 10 mm/mV).

After CTI ablation, the standard 12-lead ECGs of patients in sinus rhythm was studied by two independent observers. Advanced interatrial block (aIAB) was defined as P-wave duration > 120 ms with a biphasic morphology in inferior leads (Bayes de Luna et al., 2012; Figure 2). For patients without a bundle branch block, fragmented QRS (fQRS) complex was defined as an abnormal R′-wave notching in the nadir of S wave or the presence of more than one R′ in at least two consecutive leads. For patients with bundle branch block (QRS duration > 120 ms), fQRS complex was defined as more than two R′ or >2 notches in R wave or >2 notches in the nadir of S wave in at least two consecutive leads (Das and Zipes, 2009).

### Periprocedural Anticoagulation Strategy

All patients received oral anticoagulants (OACs) (e.g., warfarin, dabigatran, or rivaroxaban) for at least 3 weeks before ablation and continued for at least 1 month after the procedure. The international normalized ratio was maintained between 2 and 3 if the patient received warfarin. After 1 month, OAC was discontinued in asymptomatic patients whose ECG or Holter monitoring maintained sinus rhythm without AFL/AF recurrence. OAC was restarted in patients who had AFL/AF recurrence during the follow-up.

### Follow-up of Patients

No AADs were prescribed after the procedure. All patients underwent continuous ECG monitoring for at least 24 h until discharge from hospital, and they were followed up at 1, 3, 6, 9, and 12 months after the procedure and every 6 months thereafter. During each follow-up or if the patient was symptomatic, 12-lead ECG or 24-h Holter recordings were obtained to monitor rhythm status. Outpatient records, diagnoses, and subsequent hospital admissions were collected using electronic medical recording system and telephone interviews. New-onset AF was defined as any episode of AF lasting ≥30 s documented by 12-lead ECG or 24-h Holter monitoring (Poty et al., 1996). The clinical endpoint was new-onset AF during follow-up. The time interval was determined from ablation procedure to new occurrence of AF.

### Statistical Analysis

Continuous variables were expressed as mean ± standard deviation (SD) and compared using two-tailed *t*-test, while categorical variables were expressed as counts and percentage and compared using the Chi-square test or Fisher’s exact test when appropriate. The k statistic was used to evaluate the interobserver agreement (IOA) for the variables measured from the ECG. A receiver operating characteristic (ROC) curve with the area under the curve (AUC) was generated, and the optimal cut-off value of the left atrial diameter (LAD) for predicting new-onset AF was obtained. Cox regression modeling was used to determine risk factors for new-onset AF after typical AFL ablation. Variables with statistical significance (*P*-value < 0.1) in univariable regression models were included in the multivariable regression model using “forward LR” method to determine their significance after adjustment for potential confounders. Kaplan–Meier curves and log-rank tests were performed to compare AF-free survival. All statistical tests were two-sided, and statistical significance was defined as *P*-value < 0.05. All analyses were performed using the SPSS 24.0 software (SPSS Inc., Chicago, IL, United States).

## Results

A total of 191 patients were enrolled in the final analysis. Among them, the mean age was 59.0 ± 13.8 years, and the majority were males (86.4%). The baseline characteristics in detail were shown in Table 1. The bidirectional CTI block was successfully achieved without ablation complication in all 191 patients. After CTI ablation, a combination of atrial burst pacing and isoproterenol infusion was performed in eight patients, but none of them was induced with any atrial arrhythmia.

**TABLE 1:** Clinical characteristics of the study population.

Variable	Total	AF	Non-AF	*P*
	*N* = 191	*N* = 47	*N* = 144	
Age (year)	59.0 ± 13.8	60.0 ± 12.1	58.7 ± 14.3	0.772
Male (%)	165 (86.4)	40 (85.1)	125 (86.8)	0.768
Body mass index (kg/m^2^)	25.6 ± 3.5	25.3 ± 3.2	25.7 ± 3.6	0.945
New-onset AF (%)	47 (24.6)	–	–	–
**Comorbidities**				
Hypertension (%)	77 (40.3)	14 (29.8)	63 (43.8)	0.090
Diabetes mellitus (%)	37 (19.4)	8 (17.0)	29 (20.1)	0.639
Obstructive sleep apnea (%)	9 (4.7)	6 (12.8)	3 (2.1)	0.009
Ischemic heart disease (%)	15 (7.9)	3 (6.4)	12 (8.3)	0.905
Congestive heart failure (%)	4 (2.1)	1 (2.1)	3 (2.1)	1.000
Thyroid disease*	4 (2.1)	2 (4.3)	2 (1.4)	0.545
Previous stroke/TIA	4 (2.1)	2 (4.3)	2 (1.4)	0.545
**Electrocardiographic parameters**				
P-wave duration (ms)	127.7 ± 19.4	128.3 ± 18.3	127.5 ± 19.8	0.982
Right bundle branch block (%)	30 (15.7)	8 (17.0)	22 (15.3)	0.775
Left bundle branch block (%)	2 (1.0)	0 (0)	2 (1.4)	1.000
QTc interval (ms)	432.6 ± 40.2	433.6 ± 44.8	432.2 ± 38.7	0.779
Fragmented QRS complex (%)	32 (16.8)	12 (25.5)	20 (13.9)	0.063
Advanced interatrial block (%)	19 (9.9)	13 (27.7)	6 (4.2)	<0.001
**Echocardiographic parameters**				
LVEF (%)	58.7 ± 8.2	58.1 ± 8.1	58.9 ± 8.2	0.448
Left atrial diameter (mm)	39.4 ± 5.5	43.5 ± 4.7	38.1 ± 5.1	<0.001
LAD > 42mm (%)	58 (30.4)	25 (53.2)	33 (22.9)	<0.001
Enlarged right atrium (%)	35 (18.3)	12 (25.5)	23 (16.0)	0.141
CHA_2_DS_2_-Vasc score	1.4 ± 1.4	1.7 ± 1.7	1.3 ± 1.2	0.262
**Concomitant drug use**				
ACEi/ARB (%)	61 (31.9)	10 (21.3)	51 (35.4)	0.071
β-Blockers (%)	112 (58.6)	26 (55.3)	86 (59.7)	0.595
Follow-up (months)	40.2 ± 23.3	25.3 ± 24.2	45.0 ± 21.0	<0.001

*Data are presented as mean ± SD or n (%).*

*ACEi, angiotensin-converting enzyme inhibitor; AF, atrial fibrillation; ARB, angiotensin receptor blocker; LAD, left atrial diameter; LVEF, left ventricular ejection fraction; TIA, transient ischemic attack.*

*All the four patients with history of hypothyroidism had normal level of thyroid function at present.

No patient was lost to follow-up in this study. Twelve patients (6.3%) had CTI-dependent AFL recurrence at an average of 8.6 ± 7.3 months after the procedure, and seven of them had recurrence within the first year (4.1 ± 2.7 months) after ablation. Of the 12 patients, 11 patients underwent repeat CTI ablation successfully and 3 patients eventually developed AF after repeat ablation during the follow-up.

Of the 47 patients (24.6%) developing AF during a follow-up period of 3.3 ± 1.9 years, 36.2% (18/47) were documented within 1 year after procedure and 43.6% (20/47) more than 2 years after procedure. Among new AF occurrences, 93.6% (44/47) were detected by ECG or Holter monitoring and the rest 6.4% (3/47) by clinical follow-up only. Patients with obstructive sleep apnea (OSA) (12.8 vs. 2.1%, *P* < 0.001), aIAB (27.7 vs. 4.2%, *P* < 0.001), and larger LAD (43.5 ± 4.7 vs. 38.1 ± 5.1 mm, *P* < 0.001) had higher incidence of new-onset AF (Table 1).

**FIGURE 3:**
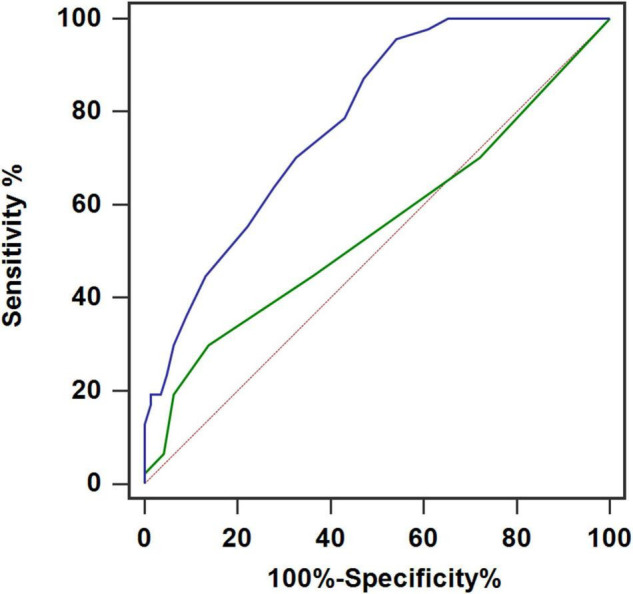
Receiver operating characteristic curves for prediction of atrial fibrillation with left atrial diameter (LAD) (blue) and CHA_2_DS_2_-VASc score (green). The cut-off value of LAD was 42 mm with area under the curve (AUC) of 0.781 (sensitivity of 95.7% and specificity of 45.8%). The cut-off value of CHA_2_DS_2_-VASc score was 2 with AUC of 0.550 (sensitivity of 29.8% and specificity of 86.1%).

**FIGURE 4:**
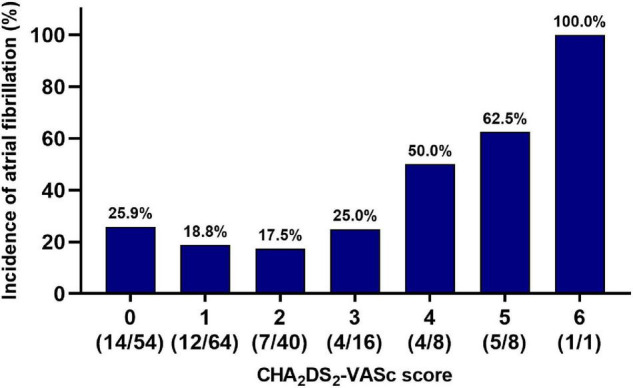
Incidence of new-onset atrial fibrillation and CHA_2_DS_2_-VASc score. The incidence of new-onset AF increased with an increase of CHA_2_DS_2_-VASc score, especially in patients with CHA_2_DS_2_-VASc > 2 (*P* = 0.046).

The ROC curves based on AF occurrence indicated that the cut-off values of LAD and CHA_2_DS_2_-VASc score were 42 mm and 2, with AUC of 0.781 and 0.550, respectively (Figure 3). Patients with LAD > 42 mm (58/191, 30.4%) had longer P-wave duration (132.4 ± 19.9 vs. 125.7 ± 18.9 ms, *P* = 0.038), more aIAB (22.4 vs. 4.5%, *P* < 0.001), and higher AF recurrence (43.1 vs. 16.5%, *P* < 0.001). For patients with CHA_2_DS_2_-VASc score > 2, advanced age (70.0 ± 12.3 vs. 56.8 ± 13.0, *P* < 0.001), hypertension (57.6 vs. 36.7%, *P* = 0.026), diabetes (54.5 vs. 12.0%, *P* < 0.001), and congestive heart failure (3.0 vs. 1.9%, *P* < 0.001) were common, and these patients were more vulnerable to AF (42.4 vs. 20.9%, *P* = 0.009) (Table 2 and Figure 4).

**TABLE 2:** Comparison between patients grouped by aIAB, LAD, and CHA_2_DS_2_-Vasc score.

Variable	aIAB+	aIAB−	*P* *	LAD > 42mm	LAD ≤ 42mm	*P* ^§^	CHA_2_DS_2_-Vasc > 2	CHA_2_DS_2_-Vasc ≤ 2	*P* ^#^
	*N* = 19	*N* = 172		*N* = 58	*N* = 133		*N* = 33	*N* = 158	
Age (year)	64.4 ± 13.5	58.4 ± 13.8	0.055	59.0 ± 13.7	59.0 ± 13.9	0.894	70.0 ± 12.3	56.8 ± 13.0	<0.001
Male (%)	14 (73.7)	151 (87.8)	0.177	51 (87.9)	114 (85.7)	0.681	29 (87.9)	136 (86.1)	0.784
BMI (kg/m^2^)	26.4 ± 4.0	25.5 ± 3.4	0.207	26.5 ± 3.6	25.2 ± 3.4	0.059	26.0 ± 3.0	25.5 ± 3.6	0.238
New-onset AF (%)	13 (68.4)	34 (19.8)	<0.001	25 (43.1)	22 (16.5)	<0.001	14 (42.4)	33 (20.9)	0.009
**Comorbidities**									
Hypertension (%)	9 (47.4)	68 (39.5)	0.509	27 (46.6)	50 (37.6)	0.246	19 (57.6)	58 (36.7)	0.026
Diabetes (%)	5 (26.3)	32 (18.6)	0.616	13 (22.4)	24 (18.0)	0.482	18 (54.5)	19 (12.0)	<0.001
OSA (%)	3 (15.8)	6 (3.5)	0.067	3 (5.2)	6 (4.5)	1.000	3 (9.1)	6 (3.8)	0.393
IHD (%)	3 (15.8)	12 (7.0)	0.365	6 (10.3)	9 (6.8)	0.580	5 (15.2)	10 (6.3)	0.175
CHF (%)	0 (0)	4 (2.3)	1.000	3 (5.2)	1 (0.8)	0.158	1 (3.0)	3 (1.9)	<0.001
Previous stroke/TIA (%)	0 (0)	4 (2.3)	1.000	1 (1.7)	3 (2.3)	1.000	2 (6.1)	2 (1.3)	0.280
Thyroid disease^†^ (%)	2 (10.5)	2 (1.2)	0.063	1 (1.7)	3 (2.3)	1.000	2 (6.1)	2 (1.3)	0.280
**Electrocardiographic parameters**									
P-wave duration (ms)	137.7 ± 15.9	126.6 ± 19.4	0.026	132.4 ± 19.9	125.7 ± 18.9	0.038	129.8 ± 21.4	127.4 ± 19.0	0.367
RBBB (%)	2 (10.5)	28 (16.3)	0.748	7 (12.1)	23 (17.3)	0.362	6 (18.2)	24 (15.2)	0.667
LBBB (%)	0 (0)	2 (1.2)	1.000	1 (1.7)	1 (0.8)	1.000	1 (3.0)	1 (0.6)	0.772
QTc interval (ms)	440.4 ± 41.0	431.7 ± 40.1	0.468	439.3 ± 46.8	429.6 ± 36.7	0.168	438.7 ± 41.1	431.3 ± 40.0	0.406
fQRS complex (%)	5 (26.3)	27 (15.7)	0.394	12 (20.7)	20 (15.0)	0.336	8 (24.2)	24 (15.2)	0.205
aIAB (%)	–	–	–	13 (22.4)	6 (4.5)	<0.001	3 (9.1)	16 (10.1)	0.857
**Echocardiographic parameters**									
LVEF (%)	59.4 ± 6.9	58.6 ± 8.3	0.662	56.4 ± 11.2	59.7 ± 6.3	0.174	59.2 ± 8.0	58.6 ± 8.2	0.544
LAD (mm)	44.7 ± 5.9	38.9 ± 5.2	<0.001	45.8 ± 3.1	36.7 ± 3.8	<0.001	41.0 ± 5.0	39.1 ± 5.6	0.088
LAD > 42 mm (%)	13 (68.4)	45 (26.2)	<0.001	–	–	–	11 (33.3)	47 (29.7)	0.684
Enlarged RA (%)	4 (21.1)	31 (18.0)	0.991	15 (25.9)	20 (15.0)	0.075	10 (30.3)	25 (15.8)	0.051
CHA_2_DS_2_-Vasc score	1.8 ± 1.7	1.4 ± 1.3	0.253	1.5 ± 1.3	1.4 ± 1.4	0.323	3.8 ± 1.0	1.0 ± 0.8	<0.001
**Concomitant drug use**									
ACEi/ARB (%)	7 (36.8)	54 (31.4)	0.629	24 (41.4)	37 (27.8)	0.065	15 (45.5)	46 (29.1)	0.067
β-Blockers (%)	11 (57.9)	101 (58.7)	0.945	40 (69.0)	72 (54.1)	0.056	21 (63.6)	91 (57.6)	0.522
Follow-up (months)	42.3 ± 30.0	39.9 ± 22.6	0.673	36.6 ± 23.8	41.7 ± 23.0	0.142	36.8 ± 24.1	40.9 ± 23.2	0.402

*Data are presented as mean ± SD or n (%).*

*ACEi, angiotensin-converting enzyme inhibitor; AF, atrial fibrillation; aIAB, advanced interatrial block; ARB, angiotensin receptor blocker; BMI, body mass index; CHF, congestive heart failure; fQRS, fragmented QRS; IHD, ischemic heart disease; LAD, left atrial diameter; LBBB, left bundle branch block; LVEF, left ventricular ejection fraction; OSA, obstructive sleep apnea; RA, right atrium; RBBB, right bundle branch block; TIA, transient ischemic attack.*

*P refers to comparison between patients with and without advanced interatrial block.

*^§^ P refers to comparison between patients with LAD > 42 mm and with LAD ≤ 42 mm.*

*^#^P refers to comparison between patients with CHA_2_DS_2_-Vasc > 2 and with CHA_2_DS_2_-Vasc ≤ 2.*

*^†^All the four patients with history of hypothyroidism had normal level of thyroid function at present.*

The *k* value that evaluated IOA for aIAB between the two cardiologists was 0.90. The presence of aIAB was observed in 19 of all 191 patients (9.9%). Patients with aIAB had longer P-wave duration (137.7 ± 15.9 vs. 126.6 ± 19.4 ms, *P* = 0.026), larger LAD (44.7 ± 5.9 vs. 38.9 ± 5.2 mm, *P* < 0.001), and higher incidence of AF (68.4 vs. 19.8%, *P* < 0.001) comparing to those without aIAB (Table 2).

**FIGURE 5:**
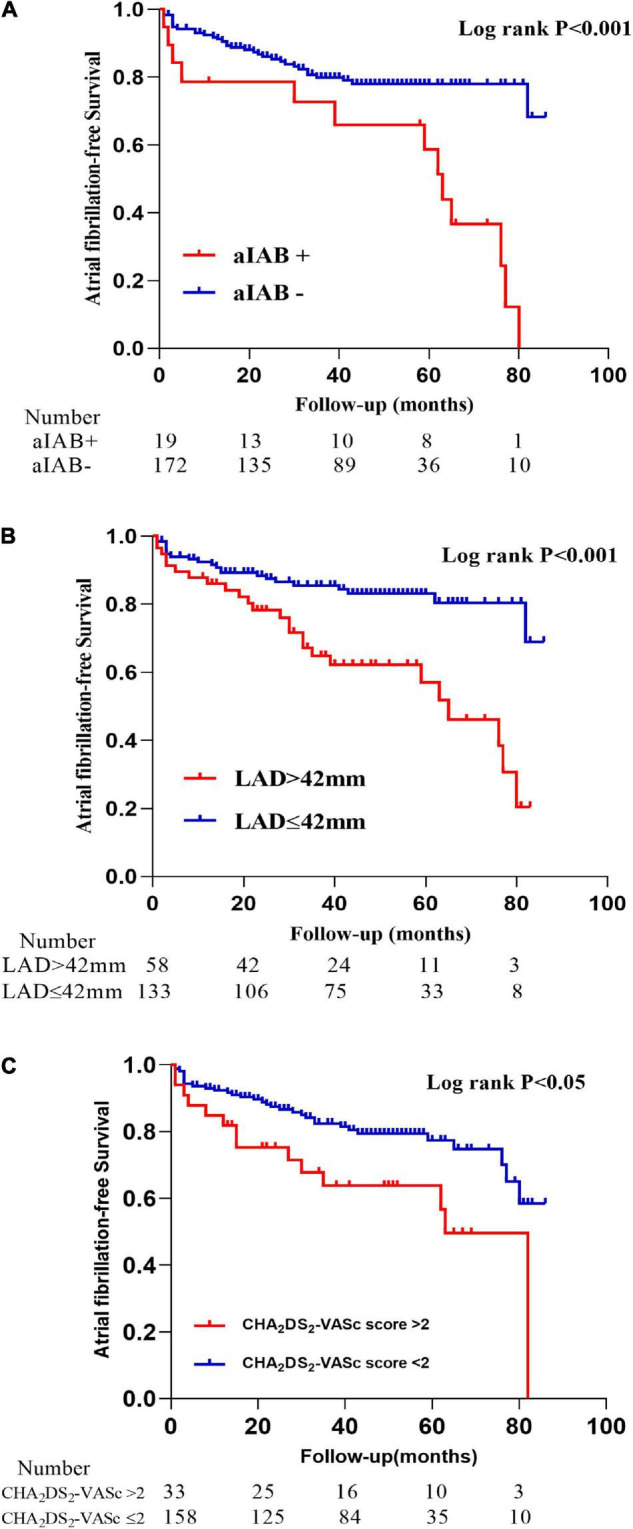
Kaplan–Meier curves of atrial fibrillation-free survival in patients grouped by advanced interatrial block **(A)**, left atrial diameter **(B)**, and CHA_2_DS_2_-VASc score **(C)**.

As shown in Kaplan–Meier curves (Figure 5), patients with aIAB, LAD > 42 mm, or higher CHA_2_DS_2_-VASc score had higher incidence of AF during the follow-up. Based on prior reports (Ellis et al., 2007; Voight et al., 2014; Celikyurt et al., 2017; Fujimoto et al., 2020) and clinical relevance, potential confounders were selected into the Cox regression model to determine risk factors for new-onset AF. The univariable analysis revealed that patients with OSA [hazard ratio (HR) 5.113, 95% confidence interval (CI) 2.143–12.196, *P* < 0.001], aIAB (HR 3.485, 95% CI 1.824–6.656, *P* < 0.001), LAD > 42 mm (HR 2.910, 95% CI 1.637–5.171, *P* < 0.001), and CHA_2_DS_2_-VASc > 2 (HR 2.287, 95% CI 1.221–4.287, *P* = 0.010) were at increasing risk of AF occurrence after CTI ablation. The multivariable Cox regression analysis revealed that OSA (HR 3.734, 95% CI 1.470–9.484, *P* = 0.006), aIAB (HR 2.034, 95% CI 1.017–4.067, *P* = 0.045), LAD > 42 mm (HR 2.710, 95% CI 1.478–4.969, *P* = 0.001), and CHA_2_DS_2_-VASc > 2 (HR 2.123, 95% CI 1.118–4.034, *P* = 0.021) were independent risk factors of new-onset AF after CTI ablation (Table 3).

**TABLE 3:** Univariable and multivariable Cox regression analysis for atrial fibrillation occurrence.

		Univariable analysis			Multivariable analysis	
Variable	HR	95% CI	*P*	HR	95% CI	*P*
P-wave duration	1.002	0.989–1.016	0.732			
aIAB	3.485	1.824–6.656	<0.001	2.034	1.017–4.067	0.045
LAD > 42 mm	2.910	1.637–5.171	<0.001	2.710	1.478–4.969	0.001
Fragmented QRS	1.652	0.854–3.192	0.136			
QTc interval	1.002	0.995–1.009	0.585			
Hypertension	0.735	0.391–1.380	0.338			
Obstructive sleep apnea	5.113	2.143–12.196	<0.001	3.734	1.470–9.484	0.006
Body mass index	0.391	0.871–1.055	0.391			
RA enlarged	1.114	0.570–2.174	0.753			
CHA_2_DS_2_-Vasc > 2	2.287	1.221–4.287	0.010	2.123	1.118–4.034	0.021

*aIAB, advanced interatrial block; CI, confidence interval; HR, hazard ratio; LAD, left atrial diameter; RA, right atrium.*

## Discussion

The main findings of the current study included that (a) among patients with typical AFL without history of AF, nearly a quarter of them developed new-onset AF after CTI ablation and (b) OSA, aIAB, higher LAD, and CHA_2_DS_2_-VASc score were independent risk factors for new-onset AF after the procedure.

### Atrial Flutter Recurrence

In present study, the average time interval between CTI ablation and AFL recurrence was over 1 year, with the interval ranging from 3 to 74 months. The rate of AFL recurrence was 6.3% and the AFL recurrence-free rate was >90%, which was consistent with reports of previous studies (Chinitz et al., 2007; Chen et al., 2015).

### Atrial Fibrillation Occurrence After Cavotricuspid Isthmus Ablation

For patients with AFL undergoing CTI ablation, the occurrence of AF has always been a concern in rhythm management after the procedure. Although the mechanism of AF development after AFL ablation has not been clearly characterized, the occurrence of these two atrial arrhythmias shares similar atrial substrate and electrophysiological triggers. Ectopic beats and focal activation from atrium or pulmonary veins lead to AF as well as the conversion from AFL to AF (Hsieh et al., 2001; Delise et al., 2006).

In present study, 24.6% of all patients developed new-onset AF in a mean follow-up time of 3.3 years after CTI ablation. This result was similar to the incidence of 25% reported by Voight et al. (2014) and 22% reported by Celikyurt et al. (2017). However, higher incidence of AF after AFL ablation was observed in some studies. Using implanted loop recorders to monitor heart rhythm, Mittal et al. (2013) reported that nearly 50% of enrolled patients developed AF after AFL ablation. Even higher incidence (82%) was observed in study from Ellis et al. (2007). Part of reason for the high incidence of AF was that patients with more complicated comorbidities were enrolled in their study. Besides, a longer period of follow-up (89 months) also allowed the progression of AF, which contributes to the higher incidence.

All patients in this study underwent CTI ablation alone, and nearly 75% of them were free from atrial arrhythmia during follow-up. In view of the risk of complications, sequential iatrogenic arrhythmia and uncertain benefit of preventing AF, prophylactic AF ablation including pulmonary vein isolation (PVI) was not routinely performed in this study, although the pulmonary vein was confirmed to have an essential role in occurrence of AF in patients with AFL (Steinberg et al., 2014; Schneider et al., 2015). Some reports (Steinberg et al., 2014; Mohanty et al., 2015; Koerber et al., 2019) demonstrated that for typical AFL patients without history of AF, CTI ablation combined with prophylactic PVI resulted in a substantial reduction in new-onset AF after procedure. However, the risk/benefit ratio of prophylactic PVI still remained controversial. Gula et al. (2016) reported greater procedural risk and higher cost for patients who underwent ablation of CTI + PVI for isolated AFL. In current study, it was confirmed that patients with OSA, aIAB, higher LAD (>42 mm), and CHA_2_DS_2_-VASc score (>2) were at significantly increasing risk of developing new-onset AF after CTI ablation for typical AFL. For these patients, active prophylactic measures brought more benefits in preventing AF occurrence, and it might be reasonable to perform PVI combined with CTI ablation. More studies evaluating the risk/benefit ratio of prophylactic PVI combined with CTI ablation were needed to confirm this assumption.

### Risk Factors for New-Onset Atrial Fibrillation

Previous evidence (Gottlieb et al., 2010; Redline et al., 2010) had shown that sleep disordered breathing increased the incidence of AF. As expected, OSA was identified as an independent risk factor for new-onset AF after ablation for typical AFL. Multiple mechanisms including atrial stretch, autonomic dysregulation, and abnormalities of gas exchange were raised to explain the relationship between AF and OSA (Nalliah et al., 2016). Furthermore, Nalliah et al. (2021) evaluated the left atrial substrate of AF patients with history of OSA by means of high-density mapping, and they found that high severity of OSA correlated with increasingly remodeled left atria, which presented with lower bipolar voltage and greater voltage/conduction heterogeneity. The electroanatomical alternations significantly promoted the development of AF. These findings suggested the importance of effective treatment of sleep disordered breathing in patients with AFL after ablation.

Although the presence of IAB is not rare in clinical work (O’Neal et al., 2016), it has been widely neglected and under-recognized (Baranchuk and Bayés de Luna, 2015). In patients with constructive abnormalities including surgical scar, fibrosis, and infarction, the conduction across the Bachmann region is delayed, while the atrial septum and/or coronary sinus plays main roll in interatrial conduction (Lemery et al., 2004; Tapanainen et al., 2009). Therefore, the left atrium is activated through the inferior interatrial pathway, and a prolonged and biphasic P wave presents in inferior leads. In this study, the presence of aIAB, which was defined as biphasic P waves with duration > 120 ms in inferior leads, was identified as an independent risk factor for new-onset AF in patients after AFL ablation. IAB was reported linking with a delayed and asynchronous activation of left atrium (Agarwal et al., 2003; Budeus et al., 2005), and it was significantly associated with AF occurrence with a HR of 2.01 (Tse et al., 2018). Also, it was a risk factor for AF recurrence after ablation (Enriquez et al., 2014; Fujimoto et al., 2018). Based on previous assumption, there might be a developing progress of atrial cardiomyopathy, starting from partial IAB in early stage to aIAB in middle stage, and eventually to developing AF. The mechanism for aIAB causing AF might involve electroanatomical remodeling, which resulted from continuous interatrial desynchrony, electrical inhomogeneity, and abnormal activation of left atrium (Fujimoto et al., 2018).

In recent years, the ability of CHA_2_DS_2_-VASc score had been beyond evaluating thromboembolic risk in patients with non-valvular AF, and it was helpful in various conditions, for example, reflecting atrial electroanatomical remodeling, which was associated with development of AF (Park et al., 2011). In present study, patients with CHA_2_DS_2_-VASc score > 2 had advance age, greater incidence of hypertension, and diabetes mellitus and larger LAD. This result implied that patients with higher CHA_2_DS_2_-VASc score were more likely to undergo electroanatomical alterations, which improved development of AF. To discriminate patients between high and low risk for AF following AFL ablation, CHA_2_DS_2_-VASc score would be a feasible choice. Besides, CHA_2_DS_2_-VASc score was useful for stratifying stroke risk following AFL ablation, and ≥2 was associated with higher risk of stroke after ablation (Jin et al., 2018). These findings combined with ours suggested a strong ability of CHA_2_DS_2_-VASc score in risk stratification and monitoring of AF, as well as prevention of thromboembolism after AFL ablation.

### Other Parameters

The fQRS complex, which reflected inhomogeneous conduction and delayed activation in myocardium (Das et al., 2007), was previously reported as a risk factor for new-onset AF after AFL ablation (Fujimoto et al., 2020). In this study, it had no significance in predicting AF after CTI ablation. The mechanism of fQRS on AF development needs further research.

In terms of the drug use, higher angiotensin-converting enzyme inhibitor (ACEi)/angiotensin receptor blocker (ARB) usage was seen in non-AF group although no significant difference was found. It suggested a potential effect of ACEi/ARBs in preventing the development of postablation AF, as previous studies (Anne et al., 2004) reported.

### Clinical Implication

Patients are still continuously at risk of AF occurrence and thromboembolic events in spite of undergoing successful ablation for typical AFL. Postablation management of high-risk patients has always been a big concern in clinical practice. In present study, the clinical value of CHA_2_DS_2_-VASc score was extended to evaluation of the risk stratification of new-onset AF. Patients with highest risk, who had OSA, aIAB, LAD > 42mm, and CHA_2_DS_2_-VASc score > 2, were strongly predisposed to AF occurrence after CTI ablation. These patients should attach importance to the likelihood of AF occurrence, and a rhythm recording device (e.g., implanted loop recorder) was necessary to detect AF occurrence as soon as possible. However, the implanted loop recorder might not be easily accessible in many medical centers. In this condition, more vigilant surveillance, such as symptom evaluation, heart structural assessment, and regular rhythm monitoring, were urgently needed for patients at high risk. Additionally, it might be reasonable to perform PVI combined with CTI ablation for high-risk patients during the AFL procedure, in order to acquire more benefits in preventing AF occurrence.

The risk stratification, which was assessed by a combination of risk factors in present study, also had guiding significance in anticoagulation strategy for patients after AFL ablation. The prevalent practice in clinical work is to withdraw OCA 1 month after AFL ablation, if the patient maintains sinus rhythm without recurrence of atrial arrhythmias (Chinitz et al., 2007; Tomson et al., 2012). Therefore, many patients, especially those with multiple comorbidities, are exposed to risk of thromboembolic complications due to a lack of risk-evaluating tools and effective anticoagulation. Previous study (Jin et al., 2018) demonstrated an increasing trend in stroke with ascending CHA_2_DS_2_-VASc score in patients following AFL ablation. Along with findings of current study, it demonstrated that for patients undergoing CTI ablation for typical AFL, it was better to identify patients at high risk in new-onset AF as the first step and then to formulate an anticoagulation strategy with a closer follow-up after the procedure.

### Limitations

Several limitations in this study should be addressed. First, the generalizability of results was limited because of the small number of subjects from only one single center. Besides, male accounted majority of the enrolled subjects (86.4%), limiting the generalizability to female. Second, although ECGs and Holter records were carefully evaluated before ablation and regularly performed during follow-up period, heart rhythm monitoring was not documented completely due to a lack of reliable recording devices such as implanted loop recorders, thus the silent AFL or AF might be neglected. Third, previous study (Joza et al., 2014) reported that the inducibility of AF after typical AFL ablation was identified as a risk factor for future AF in patients with no history of AF. However, in this study, the inducibility after CTI ablation was not routinely performed, and atrial burst pacing with infusion of isoproterenol was only attempted in few patients, which limited the evaluation of atrial burst inducibility in AF occurrence after AFL ablation. Finally, the continuation of anticoagulation after AFL ablation depended on rhythm status and AFL/AF recurrence, and it was not guided by an evaluation tool containing risk factors in present study. Also, the incidence of AF-related thromboembolism was not documented in present study. Therefore, the anticoagulation outcome in different risk stratifications of new-onset AF following AFL ablation was needed in further study.

## Data Availability Statement

The original contributions presented in the study are included in the article/supplementary material, further inquiries can be directed to the corresponding author.

## Ethics Statement

The studies involving human participants were reviewed and approved by the Ethics Committee of Fuwai Hospital. The patients/participants provided their written informed consent to participate in this study.

## Author Contributions

J-HL, H-YX, and Q-HT collected the patient data. J-HL analyzed the data and was a major contributor in writing this manuscript. Y-QC, Z-JC, X-GG, and QS took the revision of this manuscript. All authors agreed to be accountable for the content of the work.

## Conflict of Interest

The authors declare that the research was conducted in the absence of any commercial or financial relationships that could be construed as a potential conflict of interest.

## Publisher’s Note

All claims expressed in this article are solely those of the authors and do not necessarily represent those of their affiliated organizations, or those of the publisher, the editors and the reviewers. Any product that may be evaluated in this article, or claim that may be made by its manufacturer, is not guaranteed or endorsed by the publisher.
